# Assessment of Low Back Pain in Helicopter Pilots Using Electrical Bio-Impedance Technique: A Feasibility Study

**DOI:** 10.3389/fnins.2022.883348

**Published:** 2022-07-14

**Authors:** Hang Wang, Jing Dai, Chunchen Wang, Zhijun Gao, Yang Liu, Meng Dai, Zhanqi Zhao, Lin Yang, Guodong Tan

**Affiliations:** ^1^Department of Aerospace Medicine, Fourth Military Medical University, Xi’an, China; ^2^Department of Biomedical Engineering, Fourth Military Medical University, Xi’an, China; ^3^Institute of Technical Medicine, Furtwangen University, Villingen-Schwenningen, Germany; ^4^Air Force Medical Center, Fourth Military Medical University, Beijing, China

**Keywords:** low back pain, pilot, electrical bio-impedance, state of lumbar muscle, assessment

## Abstract

Low back pain (LBP) is known to pose a serious threat to helicopter pilots. This study aimed to explore the potential of electrical bio-impedance (EBI) technique with the advantages of no radiation, non-invasiveness and low cost, which is intended to be used as a daily detection tool to assess LBP in primary aviation medical units. The LBP scales (severity) in 72 helicopter pilots were assessed using a pain questionnaire, while the bilateral impedance measurements of the lumbar muscle were carried out with a high precision EBI measurement system. Results showed that the modulus of lumbar muscle impedance increased with LBP scale whereas the phase angle decreased. For different LBP scales, significant differences were found in the modulus of lumbar muscle impedance sum on both sides (*Z*_*sum*_), as well as in the modulus and phase angle of lumbar muscle impedance difference between both sides (*Z*_*diff*_ and *ϕ*_*diff*_), respectively (*P* < 0.05). Moreover, Spearman’s correlation analysis manifested a strong correlation between *Z*_*sum*_ and LBP scale (*R* = 0.692, *P* < 0.01), an excellent correlation between *Z*_*diff*_ and LBP scale (*R* = 0.86, *P* < 0.01), and a desirable correlation between *ϕ*_*diff*_ and LBP scale (*R* = −0.858, *P* < 0.01). In addition, receiver operator characteristic analysis showed that for LBP prediction, the area under receiver operator characteristic curve of *Z*_*sum*_, *Z*_*diff*_, and *ϕ*_*diff*_ were 0.931, 0.992, and 0.965, respectively. These findings demonstrated that EBI could sensitively and accurately detect the state of lumbar muscle associated with LBP, which might be the potential tool for daily detection of LBP in primary aviation medical units.

## Introduction

Low back pain (LBP) is a recurrent or sometimes persistent disorder defined as the pain localized between the 12th rib and the inferior gluteal folds, which has been one of the most common musculoskeletal diseases worldwide (Krismer and van Tulder, 2007; Wu et al., 2020). For helicopter pilots, due to prolonged exposure to whole-body vibration of high-intensity and confined sitting posture, they complain about LBP frequently (Posch et al., 2019; da Silva, 2020). LBP poses a serious threat to helicopter pilots because it can impair the operational capabilities closely related to flight safety, such as attention, motion control and postural stability (Harrison et al., 2009; Gaydos, 2012). To prevent LBP and alleviate its adverse effects, a comprehensive set of solutions, including flight-specific exercises, the use of lumbar support as well ergonomic cockpit and seat, have been applied for helicopter pilots (Gaydos, 2012; Murray et al., 2015; Andersen et al., 2017). Unfortunately, the incidence of LBP among helicopter pilots has remained high as a result of inevitable vibration and poor sitting posture. A recent epidemiological survey reported that the 3-month and 12-month prevalence of LBP for military helicopter pilots was as high as 42.3 and 48.1% (Posch et al., 2019), respectively. Meanwhile, aviation medicine research showed that early detection of LBP and timely implementation of medical intervention prior to the observation of apparent symptoms, such as physiotherapy and neuromuscular electrical stimulation, could particularly be beneficial to the health and well-being of pilots (Palmer and Bovenzi, 2015; Alrwaily, 2017; Marins et al., 2020). Additionally, objective assessment of LBP severity is helpful for implementing appropriate and specific treatment. Therefore, objective detection and evaluation of LBF is of critical importance to minimize the influences of LBP on helicopter pilots’ health and their flight performance.

Numerous studies have shown that the lumbar muscle dysfunction including the loss of strength and endurance of muscle is considered predictive for the development of LBP. The existing evaluation methods in clinical practice involves surface electromyogram (sEMG) and functional imaging. The parameters in frequency and time domain of sEMG signal can reflect the lumbar muscle function (Villafane et al., 2016; Rose-Dulcina et al., 2020; Arvanitidis et al., 2021), but the sEMG characteristics of the lumbar muscle associated with LBP have not been systematically clarified (Wang et al., 2019). Although functional imaging such as X-ray, CT and MRI can accurately diagnose the obvious changes in muscle structure and state, these imaging modalities might be not suitable as the daily detection tool in primary medical units to evaluate LBF for consideration of radiation and cost of equipment (Ching et al., 2013). Additionally, some novel methods such as sonomyography and acoustic myography can reflect the muscle pain by describing the muscle structure and morphology, but their clinical effectiveness needs to be further verified (Huang et al., 2007; Harrison, 2018; Zhang et al., 2022). Moreover, although LBP can be assessed by subjective evaluation, this practice may not reflect the true state of lumbar muscle because there exist differences in pain tolerance among people. Thus, it is still necessary to explore a simple and effective method for daily LBP detection in primary medical units.

Electrical bio-impedance (EBI) is a relatively new technique that can provide information on physiological and pathological states of human tissues (Abe et al., 2021; Al-harosh et al., 2022). In EBI, a small amount of electrical current is injected into the targeted area of the body through surface electrodes and simultaneously the resulting voltage across the internal tissues are measured to calculate the electrical impedance of the tissue. With the unique advantages of no radiation, non-invasiveness, easy operation and portability, EBI has been widely employed in biomedical applications, especially in the assessment of the state of human tissues, such as brain edema and inflammation (Lingwood et al., 2003; Mortreux et al., 2019; Dai et al., 2020; Al-harosh et al., 2022). Moreover, previous studies have shown that the impedance characteristics of muscle is highly related to muscle state, and thus in theory, EBI has potential to detect LBP of helicopter pilots by assessing the state of lumbar muscle (Ching et al., 2013; Wang et al., 2019).

To date, several studies have explored the ability of EBI to detect LBP. Wang et al. (2019) found that impedance phase (at 100 kHz) of lumbosacral paraspinal muscle in LBP patients was lower than that in healthy individuals. They also observed significantly increased difference in impedance between both sides of the spine in LBP patients. Ching et al. (2013) found significant differences in impedance properties of lumbar paraspinal muscles between acute LBF patients and healthy subjects. Fujimoto et al. (2019) compared the measurements of dual-energy X-ray absorptiometry (DXA) and bio-impedance analysis among LBP patients and concluded that EBI and DXA were significantly correlated in both female and male patients. Other studies also reported the difference in electrical impedance between LBP patients and healthy individuals (Fujimoto et al., 2018; Rutkove et al., 2019). These previous studies demonstrated the high sensitivity of EBI to lumbar muscle dysfunction caused by LBP; however, these results seemed insufficient to support direct use of EBI to detect LBP in helicopter pilots. First, the subjects studied in previous literature were patients, rather than particular group of helicopter pilots. Second, previous studies did not perform any quantitative pain rating for LBP so that no relationship between the severity of LBP and muscle impedance could be established.

In this study, the severity (scale) of LBP in 72 helicopter pilots was determined and impedance measurements of the lumbar muscle were carried out. Next, the impedance characteristics of lumbar muscle with different LBP scales were compared. Finally, the correlation between muscle impedance and LBP scales was established to explore the feasibility of using EBI to evaluate the lumbar muscle dysfunction caused by LBP.

## Materials and Methods

### Participants and Ethics Statement

A total of 92 Chinese male helicopter pilots were invited to participate in the present study. All invited participants were informed of the study protocol. Finally, 72 pilots accepted the invitation and were enrolled in this study. Inclusion criteria were: (1) maintaining operational flight status at enrollment, (2) having the flying experience of more than one year, and (3) having no history of spinal fractures or surgery. Exclusion criterion was: receiving the treatment or intervention for LBP prior to the study.

All participants signed the written consent form prior to the study. This study was approved by the Ethics Committee of Fourth Military Medical University (No.KY20163064-1) and carried out in accordance with relevant regulations and guidelines.

### Questionnaire and Implementation

The Chinese version of Oswestry Disability Index (ODI), one of the most commonly used patient-reported outcome measures for individuals with LBP in clinical setting (Chow and Chan, 2005), was adopted to evaluate the severity of LBP for each pilot. ODI consists of ten sections to assess the extent of LBP according to various activities of daily life, including personal care, lifting, walking, sitting, standing, sleeping, sex (if applicable), social and travel. Each section has 6 scales, corresponding to 0–5 points. The higher the score, the more severe the dysfunction. Total score summarizes the scores in all sections and is normalized according to Eq. 1.

Total score = [(total score)/(number of items completed by subjects × 5)] × 100 (Krismer and van Tulder, 2007).

Scores are stratified into severity: 0–20%, minimal disability; 21–40%, moderate disability; 41–60%, severe disability; 61–80%, crippling back pain; 81–100%, these patients are either bed-bound or have an exaggeration of their symptoms.

In this study, we defined an LBP scale, ranging from 1 to 5, as no pain, mild dysfunction, moderate dysfunction, severe dysfunction and complete dysfunction, respectively. The LBP score is a linearly discretized variation of the total score. Table 1 shows the corresponding relationship between total scores, ODI scales and LBP scales. It should be stated that the minimal disability in the ODI scales was divided into no pain and mild pain in the LBP scales, so that the differences could be compared in lumbar muscle impedances between the cases of no pain and pain. Additionally, among all pilots, none scored more than 60, which meant that no one had crippling back pain or was bed-bound; therefore, these two ODI scales were integrated as complete dysfunction in the LBP scales.

**TABLE 1:** The corresponding relationship between ODI scale and LBP scales.

Scores	ODI scales	LBP scales
0%	minimal	1 (no pain)
1–20%	minimal	2 (mild)
21–40%	moderate	3 (moderate)
41–60%	severe	4 (severe)
61–80%	crippling	5 (complete dysfunction)
81–100%	bed-bound or exaggeration	5 (complete dysfunction)

### Bioimpedance Measurement

**FIGURE 1:**
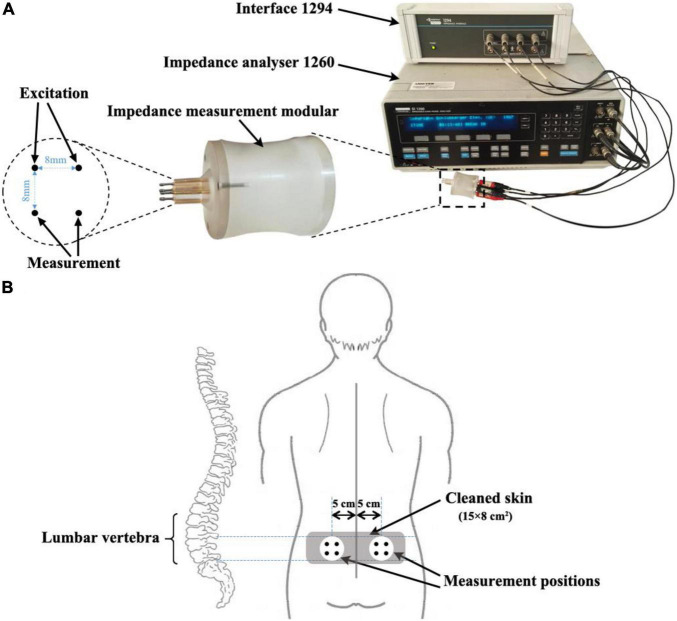
Impedance measurement system and schematic diagram of measurement positions. **(A)** Impedance measurement system consists of an impedance analyzer (Solartron 1260), a measurement interface (Solartron 1294) and a measurement module developed by our group. **(B)** Schematic diagram of measurement positions: the impedance measurement module was placed between L4-5 with its center 5 cm from each side of the spine.

Impedance measurement of lumbar muscle on both sides of the spine was performed. The bioimpedance measurement system consists of a Solartron 1260 impedance analyzer and a Solartron 1294 interface (Solartron Analytical, Farnborough, United Kingdom), which can operate at the frequency range from 10 Hz to 1 MHz with the accuracy of better than 0.01%. In this study, the current of 1 mA at 50 kHz was applied because the characteristic frequency of impedance property of muscle tissue was around 50 kHz (Bartels et al., 2015; Pandeya et al., 2021). In order to reduce the effect of electrode-skin contact impedance, the four electrode strategy was adopted (Yang et al., 2016, 2017b). To facilitate the implementation of *in vivo* measurements, we developed a measurement module that can conveniently convert the four electrodes of measurement system to four probes made of silver, two of which were used for excitation and the other two for measurement, as shown in Figure 1. The four probes (1 mm in diameter) are distributed at four corners of a square with a side length of 8 mm. This electrode configuration has been widely used for impedance measurement of biological tissues (Smallwood et al., 2002; Keshtkar, 2007; Laufer et al., 2010; Keshtkar et al., 2012; Bharati et al., 2013).

The impedance measurement process of lumbar paraspinal muscle was as follows. First, the participant was asked to maintain an upright sitting position. An experienced doctor (specialized in aerospace medicine) determined the region over the L4-5 paraspinal skin (approximately 15 × 8 cm^2^) and cleaned the skin with medical alcohol. Second, the impedance module was placed in the cleaned area with its center 5 cm from the right side of the spine; and the connecting line of the two excitation probes was parallel to the spine. Then impedance measurement was performed. Third, the impedance measurement on the left side was carried out subsequently at the symmetrical position of the impedance module. To minimize random error in the measurement, the impedance measurements were conducted three times on each side within 2 min. The average of the three measurements was taken as the final result.

### Statistical Analysis

The data were summarized in mean ± standard deviation. The coefficient of variation of impedance measurement was calculated for each measurement position to evaluate the measurement reliability.

The impedance modulus and phase of the lumbar muscle on left and right sides of the spine for each participant were obtained, which were denoted as *Z*_*L*_,*ϕ*_*L*_,*Z*_*R*_ and *ϕ*_*R*_, respectively. To evaluate the sensitivity of lumbar muscle impedance to LBP, the sum of impedance on both sides (i.e., *Z*_*sum*_ = *Z*_*L*_ + *Z*_*R*_ and *ϕ*_*sum*_ = *ϕ*_*L*_ + *ϕ*_*R*_) was compared between individuals with LBP (Scale 2–5) and those without LBP (Scale 1). The reason for this is that different severities of LBP affected the microstate of lumbar muscle to varying degrees. Also, the sum of impedance on both sides (*Z*_*sum*_ and *ϕ*_*sum*_) for different LBP scales was, respectively, compared. Similarly, in order to further analyze the effect of LBP on the impedance characteristic of the lumbar muscle, the impedance differences between the two sides (i.e., *Z*_*diff*_ = |*Z*_*L*_−*Z*_*R*_| and *ϕ*_*diff*_ = |*ϕ*_*L*_−*ϕ*_*R*_|) were compared between individuals with LBP (Scale 2–5) and those without LBP (Scale 1). This can be justified by the fact that the extent to which LBP affects the microstate of the lumbar muscle on the left and right side of the spine is not exactly the same. Moreover, *Z*_*diff*_ and *ϕ*_*diff*_ for different LBP scales were, respectively, compared. The statistical comparisons were carried out with the *post hoc* test after one-way analysis of variance (ANOVA).

To establish the relationship between lumbar muscle impedance and LBP, the Spearman’s correlation was utilized to assess the correlation between LBP scale and impedance characteristics (*Z*_*sum*_,*ϕ*_*sum*_,*Z*_*diff*_, and *ϕ*_*diff*_), in which all impedance characteristics and the corresponding LBP scales (Scale 1–4) were involved. To evaluate the ability of lumbar muscle impedance for detection of LBP (namely differentiation of pain from no pain), the receiver operator characteristics (ROC) analysis was employed to calculate the sensitivity, specificity, and area under the ROC curve (AUC) of impedance for LBP prediction. In the ROC analysis, all impedances and the corresponding LBP scales (Scale 1–4) were used to predict pain (Scale 2–4), i.e., the impedance corresponding to Scale 1 (no pain) was marked as 0 and the impedance corresponding to Scale 2–4 (pain) was marked as 1. SPSS 22.0 (IBM Software, Armonk, NY, United States) was utilized for statistical analysis and a *P*-value < 0.05 was considered statistically significant.

## Results

### Basic Information

#### Participant Characteristics

**FIGURE 2:**
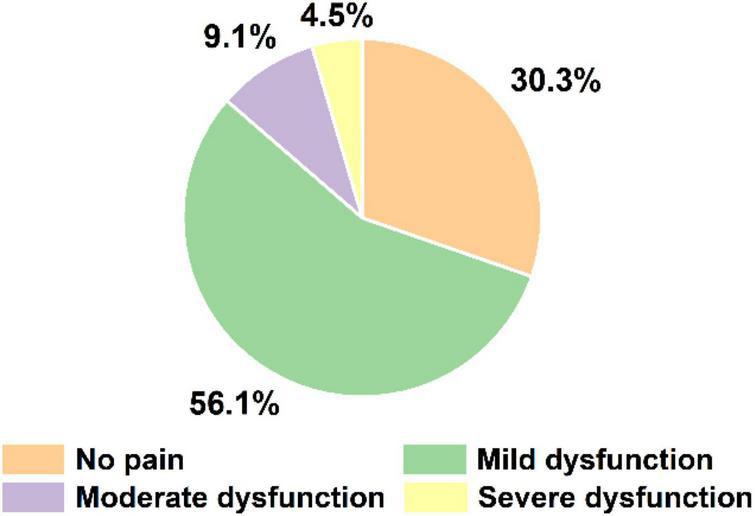
The distribution of subjects with corresponding LBP scale. Complete dysfunction was not applicable to any participants.

Participants were aged from 22 to 48 (32.49 ± 6.56) years, with a weight range from 62 to 91 (73.23 ± 6.78) kg and a height range from 167 to 185 (174.41 ± 3.90) cm. Figure 2 shows the symptoms of LBP and the ODI questionnaire results. Fifty-one participants reported the presence of LBP, 14 individuals already had moderate and severe dysfunction, but no one had complete dysfunction.

#### Variability of Impedance Measurement

**FIGURE 3:**
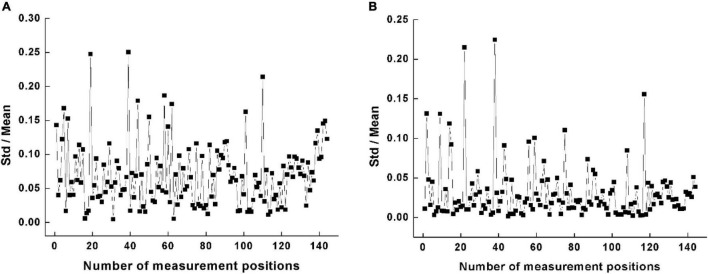
Variability of impedance measurement at all measurement positions. **(A)** Coefficient of variation for the impedance modulus. **(B)** R coefficient of variation for the impedance phase angle.

A total of 144 sets of impedance data were obtained from the lumbar muscle on both sides of the spine in 72 subjects. Figure 3 shows that the coefficient of variation for the impedance modulus and for the phase angle at all measurement positions were all less than 0.25.

### Analysis of Sensitivity of Lumbar Muscle Impedance to Low Back Pain

#### Comparison of *Z*_*sum*_ and *ϕ*_*sum*_ for Different Low Back Pain Scales

**FIGURE 4:**
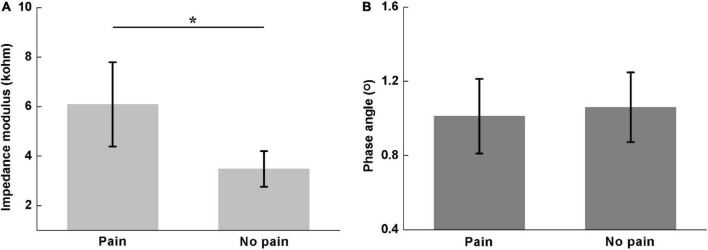
Comparisons of the sum of lumbar muscle impedance on both sides between participants with and without LBP, including **(A)** impedance modulus (pain: 6.09 ± 1.70; no pain: 3.48 ± 0.72) and **(B)** phase angle (pain: 1.01 ± 0.20; no pain: 1.06 ± 0.19). *denotes *P* < 0.05.

Figure 4 shows the comparison of the *Z*_*sum*_ and *ϕ*_*sum*_ between participants without LBP (Scale 1) and those with LBP (Scale 2–4). There was a significant difference in impedance modulus *Z*_*sum*_(pain: 6.09 ± 1.70; no pain: 3.48 ± 0.72; *P* < 0.05), whereas no significant difference was found in impedance phase angle *ϕ*_*sum*_ (pain: 1.01 ± 0.20; no pain: 1.06 ± 0.19; *P* = 0.736).

**FIGURE 5:**
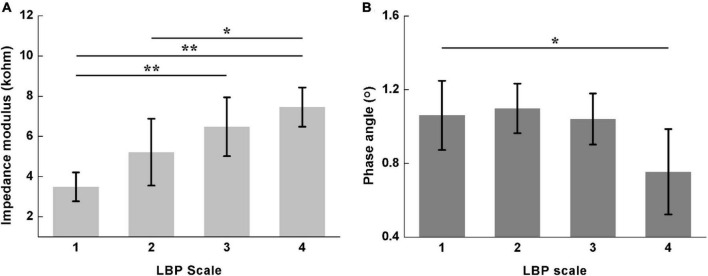
Comparisons of the sum of lumbar muscle impedance on both sides for different LBP scales, including **(A)** impedance modulus (Scale 1: 3.48 ± 0.72; Scale 2: 5.22 ± 1.67; Scale 3: 6.48 ± 1.46; Scale 4: 7.45 ± 0.98) and **(B)** phase angle (Scale 1: 1.06 ± 0.19; Scale 2: 1.10 ± 0.13; Scale 3: 1.04 ± 0.14; Scale 4: 0.76 ± 0.23). Scale 1–4 of LBP represent no pain, mild dysfunction, moderate dysfunction and severe dysfunction, respectively. *denotes *P* < 0.05 and **denotes *P* < 0.01.

Figure 5 shows the comparisons of *Z*_*sum*_ and *ϕ*_*sum*_ for different LBP scales. The *Z*_*sum*_ increased whereas the *ϕ*_*sum*_ decreased along with the LBP severity. Significant differences in *Z*_*sum*_ were found between Scale 1 and 3 (Scale 1: 3.48 ± 0.72; Scale 3: 6.48 ± 1.46; *P* < 0.01), Scale 1 and 4 (Scale 1: 3.48 ± 0.72; Scale 4: 7.45 ± 0.98; *P* < 0.01), and Scale 2 and 4 (Scale 2: 5.22 ± 1.67; Scale 4: 7.45 ± 0.98; *P* < 0.05), respectively. However, for *ϕ*_*sum*_, significant difference was only found between Scale 1 and 4 (Scale 1: 1.06 ± 0.19; Scale 4: 0.76 ± 0.23; *P* < 0.05).

#### Comparison of *Z*_*diff*_ and *ϕ*_*diff*_ for Different Low Back Pain Scales

**FIGURE 6:**
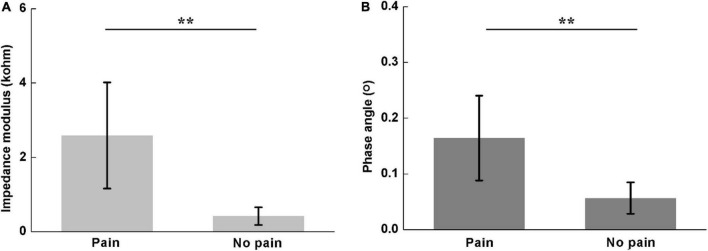
Comparisons of difference of lumbar muscle impedance on both sides between participants with and without LBP, including **(A)** impedance modulus (pain: 2.59 ± 1.42; no pain: 0.42 ± 0.24) and **(B)** phase angle (pain: 0.10 ± 0.05; no pain: 0.11 ± 0.03). **denotes *P* < 0.01.

Figure 6 shows the comparison of *Z*_*diff*_ and *ϕ*_*diff*_ between participants with and without LBP. There were significant differences in both *Z*_*diff*_(pain: 2.59 ± 1.42; no pain: 0.42 ± 0.24; *P* < 0.01) and *ϕ*_*diff*_(pain: 0.16 ± 0.08; no pain: 0.06 ± 0.03; *P* < 0.01).

**FIGURE 7:**
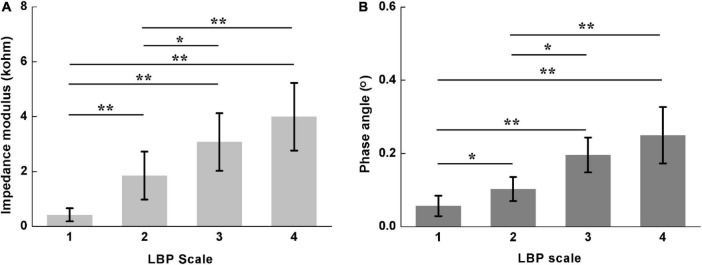
Comparison of difference of lumbar muscle impedance on both sides for different LBP scales, including **(A)** impedance modulus (Scale 1: 0.42 ± 0.24; Scale 2: 1.85 ± 0.87; Scale 3: 3.08 ± 1.05; Scale 4: 3.99 ± 1.23) and **(B)** phase angle (Scale 1: 0.06 ± 0.03; Scale 2: 0.10 ± 0.03; Scale 3: 0.20 ± 0.05; Scale 4: 0.25 ± 0.08). LBP Scale 1–4 represents no pain, mild dysfunction, moderate dysfunction and severe dysfunction, respectively. *denotes *P* < 0.05; **denotes *P* < 0.01.

Figure 7 shows the comparison of *Z*_*diff*_ and *ϕ*_*diff*_ for different LBP scales. Significant differences in *Z*_*diff*_ were found among four pain scales (Scale 1: 0.42 ± 0.24; Scale 2: 1.85 ± 0.87; Scale 3: 3.08 ± 1.05; Scale 4: 3.99 ± 1.23; *P* < 0.05), except between Scale 3 and 4 (*P* = 0.465). Similarly, significant differences were also found in *ϕ*_*diff*_ among four pain scales (Scale 1: 0.06 ± 0.03; Scale 2: 0.10 ± 0.03; Scale 3: 0.20 ± 0.05; Scale 4: 0.25 ± 0.08; *P* < 0.05), except between Scale 3 and 4 (*P* = 0.402).

### Assessment of the Relationship Between Lumbar Muscle Impedance and Low Back Pain

#### Correlation Between Lumbar Muscle Impedance and Low Back Pain Scale

The correlations between lumbar muscle impedance and LBP are presented in Table 2, in which the following significant results could be observed: a positive association between *Z*_*sum*_ and LBP scales (*R* = 0.692, *P* < 0.01), and a weak negative association between *ϕ*_*sum*_ and LBP scales (*R* = −0.281, *P* > 0.05). Also, the higher association was found between *Z*_*diff*_ and LBP scales (*R* = 0.860, *P* < 0.01), and also between *ϕ*_*diff*_ and pain scales (*R* = −0.858, *P* < 0.01).

**TABLE 2:** Correlation between lumbar muscle impedance and pain scale.

Lumbar muscle impedance		Pain scale
Sum of lumbar muscle impedance on both sides	Modulus (*Z*_*sum*_)	*R* = 0.692 *P* = 2.5133E-10**
	Phase angle (*ϕ*_*sum*_)	*R* = −0.281 *P* = 0.25
Difference of lumbar muscle impedance on both sides	Modulus (*Z*_*diff*_)	*R* = 0.860 *P* = 1.3815E-18**
	Phase angle (*ϕ*_*diff*_)	*R* = −0.858 *P* = 9.6273E-19**

**denotes P < 0.001.

#### Identification Evaluation

**FIGURE 8:**
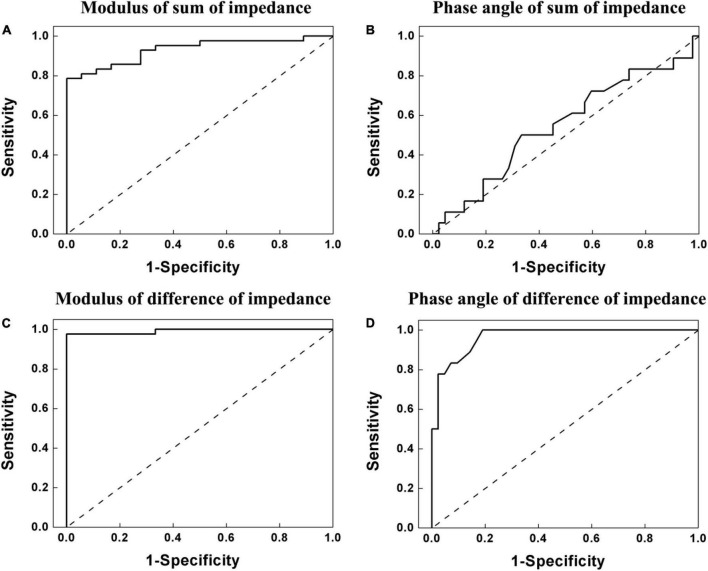
Receiver operating characteristic (ROC) curve of the lumbar muscle impedance for ability to predict pain (Scale 2, 3, and 4 in ODI). **(A)** Modulus of sum of lumbar muscle impedance on both sides. **(B)** Phase angle of sum of lumbar muscle impedance on both sides. **(C)** Modulus of difference of lumbar muscle impedance on both sides. **(D)** Phase angle of difference of lumbar muscle impedance on both sides. For modulus (*Z*_*sum*_) and phase angle (*ϕ*_*sum*_) of sum of lumbar muscle impedance on both sides, as well as modulus (*Z*_*diff*_) and phase angle (*ϕ*_*diff*_) of difference of lumbar muscle impedance on both sides, the areas under the ROC curve (AUC) of the, the were 0.931, 0.548, 0.992, and 0.965, respectively, for pain prediction (LBP Scale 2, 3, and 4). The sensitivity and specificity range from 0.501 to 0.976 and from 0.667 to 1, respectively.

Receiver operator characteristics analysis showed that AUC of *Z*_*sum*_,*ϕ*_*sum*_,*Z*_*diff*_ and *ϕ*_*diff*_ were 0.931, 0.548, 0.992, and 0.965 for pain prediction (LBP Scale 2, 3, and 4), respectively. The sensitivity and specificity ranged from 0.501 to 0.976 and from 0.667 to 1, respectively (Figure 8 and Table 3).

**TABLE 3:** Area under the receiver operator characteristic curves for ability to predict pain for lumbar muscle impedance of helicopter pilots.

	Sum of lumbar muscle impedance on both sides		Difference of lumbar muscle impedance on both sides	
	modulus	phase angle	modulus	phase angle
AUC	0.931	0.548	0.992	0.965
cut-off value	4.86 (kohm)	1.1456 (°)	0.915 (kohm)	0.1656 (°)
J-Youden	0.730	0.167	0.976	0.762
Sensitivity	0.786	0.501	0.976	0.833
Specificity	0.944	0.667	1	0.929

*AUC, area under receiver operator characteristic curve.*

## Discussion

In this study, we explored the feasibility of using EBI to assess LBP by means of measuring lumbar muscle impedance and LBP scales in 72 helicopter pilots. The correlation between impedance characteristics and LBP scales were significant.

### Summary and Explanations of Experimental Results

#### Analysis of Sensitivity of Lumbar Muscle Impedance to Low Back Pain

In this study, the modulus of lumbar muscle impedance increased with the LBP severity whereas the phase angle decreased. Also for different LBP scales, significant differences were found, respectively, in *Z*_*sum*_, as well as in *Z*_*diff*_ and *ϕ*_*diff*_. These results indicated the capability of lumbar muscle impedance to accurately reflect LBP scales, suggesting that lumbar muscle impedance should be sensitive to the state of lumbar muscle associated with LBP in helicopter pilots. Overall, our results largely agree with the previous studies. For example, Fujita et al. (2001) and Ching et al. (2013) observed that individuals with LBP had larger impedance than healthy volunteers. Dibai-Filho et al. (2018) concluded that individuals with lower pressure pain thresholds had higher impedance in the torso. However, these previous studies aimed at clinical patients, rather than the particular group of helicopter pilots. Additionally, contrary to the present study, Yamamoto et al. (2006) found a significant decrease in electrical impedance after pain provocation in patients with shoulder pain. This difference may be attributed to some practical factors, such as impedance measurement strategy. In the study of Yamamoto et al. (2006) the impedance measurement was carried out using three-electrode method with a very low frequency current (10 Hz). Considering that the electrode-skin contact impedance was much larger than the tissues impedance at low frequencies (below 1 kHz) (Rosell et al., 1988; Yang et al., 2017a), their measurement results included not only the tissue impedance between measuring electrodes, but also the electrode-skin contact impedance. To effectively eliminate the influence of electrode-skin contact impedance on the measurement results, we adopted the four-electrode approach to perform impedance measurement in this study (Bartels et al., 2015; Pandeya et al., 2021).

The difference of lumbar muscle impedance for different LBP scales could be associated with the physiological structure of muscle tissue. Due to the peculiarities of the occupation, helicopter pilots were often exposed to the vibration and fixed sitting posture for a long time, which readily caused muscle fatigue. The continuous accumulation of muscle fatigue could lead to chronic inflammation or even microvascular damage in the internal muscle tissue. Inflammation could cause two changes, which have opposite effects on the lumbar muscle impedance. On one hand, the muscle inflammation commonly resulted in local swelling because more interstitial fluid and blood at the local area were required for the facilitation of muscle repair. This increased the extracellular gaps and thus would be expected to decrease resistance (Song et al., 2018). On the other hand, the inflamed muscles were under repair and thus many proteins needed in the repairmen process were synthesized inside the muscle cells. This could cause a significant increase of intracellular resistance. However, according to the equivalent circuit of the impedance of the lumbar paraspinal muscles suggested by Yamamoto and Yamamoto (1976), the increase of intracellular resistance of inflamed muscles would increase the overall impedance of lumbar muscle tissue when the high frequency current (>1 kHz, 50 kHz in this study) was employed because it could pass through the cell membrane (Ching et al., 2013). Therefore, the modulus of lumbar muscle impedance of pilots with LBP was higher than those without, as shown in Figure 3. Moreover, as the LBP severity increased, more severe inflammation further increased the impedance modulus, as shown in Figure 4.

As for the change in the phase angle caused by LBP, the possible explanations were as follows. At the stage of muscle inflammation, the myocyte membranes remained stable or underwent little change, so the reactance of lumbar muscle impedance might not change greatly. However, due to the significant increase in the resistance of lumbar muscle impedance, the phase angle (determined by the ratio of reactance to resistance) of lumbar muscle impedance decreased (Figures 3, 4).

#### Analysis of Relationship Between Lumbar Muscle Impedance and Low Back Pain

It was found that the trend of modulus of lumbar muscle impedance was the same as that of LBP scale. By contrast, the trend of impedance phase angle was exactly the opposite to that of LBP scale. Besides, Spearman’s correlation analysis manifested that there was a strong correlation between *Z*_*sum*_ and LBP scale (*R* = 0.692, *P* < 0.01), an excellent correlation between *Z*_*diff*_ and LBP scale (*R* = 0.86, *P* < 0.01), and a desirable correlation between *ϕ*_*diff*_ and LBP scale (*R* = −0.858, *P* < 0.01). These high correlation coefficients suggested that *Z*_*sum*_,*Z*_*diff*_, and *ϕ*_*diff*_ of EBI technique are potentially useful metrics to assess LBP scale. In addition, ROC analysis showed that in pain prediction (LBP Scale 2, 3, and 4), AUC of *Z*_*sum*_,*Z*_*diff*_, and *ϕ*_*diff*_ are all above 0.93, indicating that EBI could determine the state of lumbar muscle associated with LBP.

In the present study, the correlations between *Z*_*diff*_ and LBP scales, *ϕ*_*diff*_ and LBP scales were higher than those using *Z*_*sum*_ and *ϕ*_*sum*_. This might be because the process of subtraction removed some of inter-individual variability. First, despite of strict control in the placement of measurement probes, the probe positions on each subject could not be ensured to be identical owing to the variation in individual low back sizes and shapes. It should be noted that a small error of probe position could lead to a large alteration of impedance (Dai et al., 2020). Second, there were significant differences in physical characteristics of all the subjects. For example, the weight of all the subjects widely ranged from 62 to 91 (73.23 ± 6.78) kg and their height ranged from 167 to 185 (174.41 ± 3.90) cm. These physiological differences could result in considerable variations in the distributions of electric fields for all individuals. Therefore, in the future, a standard strategy of impedance measurement as well as the reliable parameters extracted from lumbar muscle impedance should be explored to alleviate the effects of inter-individual variability, so that the quantitative relationship between lumbar muscle impedance and LBP could be established. Nevertheless, with high correlations between the EBI-based parameters and LBP scores (Table 2) and high AUC (Figure 8), it suggested that EBI technique was a potentially useful tool to assess LBP severity.

### Considerations in Assessment of Low Back Pain With Electrical Bio-Impedance in Practical Application

In this study, we performed impedance measurement by using a commercial high-accuracy impedance measurement system. This system ensured the correctness and reliability of our experimental results; however, its large size, heavy weight and high cost make it difficult to be widely used in the primary medical units. Thus, from the perspective of practical application, new impedance measurement system need to be developed. First, in order to minimize the size and weight of the measurement, highly integrated electronic components are preferred, such as the high-precision chip AD5933 specially designed for bioimpedance measurement (Yamamoto and Yamamoto, 1976). Second, for convenient use, it is best to integrate the impedance system into a chip or a circuit board to further reduce its size to embed it into the measurement module. Third, a small excitation current should be explored (1 mA was used in the present study) whether it could also obtain reliable measurements to evaluate LBP, so that a battery of small size can be used as power supply to further minimize the impedance system (Yang et al., 2021).

### Limitations and Future Work

In this study, we measured the impedance of lumbar muscle only at the very frequency of 50 kHz for the following reasons. First, in practice, for rapid assessment of LBP of pilots, the impedance at one single frequency was preferred. Second, Bartels et al. (2015) measured the impedance of muscle tissue at frequencies between 4 and 1,000 kHz and they found that the characteristic frequency of impedance property of muscle tissue was around 50 kHz. However, the use of the excitation current at a single frequency may have several limitations. First, the impedance measurement at a fixed frequency could not allow the mathematical modeling of impedance spectra properties to obtain dielectric parameters, such as characteristic frequencies within different frequency dispersions ranges in the Cole-Cole model (Tang et al., 2009; Elwakil et al., 2021). These dielectric parameters may be more useful to evaluate the state of lumbar muscle. Second, impedance information at low frequencies (<1 kHz) was not involved. In several studies it was found that impedance at low frequencies may be more sensitive to muscle state (Kumar et al., 2019a; Mortreux et al., 2019). Therefore, further studies on impedance measurement by sweeping frequency over a wide frequency range will be designed to investigate the specified dielectric parameters to assess LBP on the premise of rapid measurement and safety.

In this study, the impedance of lumbar muscle on each side of the spine was measured in a short time period of 2 min, whereas the time-varying impedance of lumbar muscle was not measured. Considering the possibility of using the time-varying impedance to reflect the development of LBP, we will design a bioimpedance system to monitor/measure time-varying impedance of lumbar muscle. Moreover, the ability of lumbar muscle impedance to detect LBP, namely differentiate pain from no pain, was preliminarily explored by performing ROC analysis. However, the impedances of lumbar muscles in only 72 helicopter pilots were collected in this study, which may not be enough for accurate prediction of pain. In future work, a larger impedance database from helicopter pilots will be established to improve the accuracy of pain prediction.

In this study, we measured the impedance of lumbar muscle and attempted to establish the correlation between impedance properties and LBP. Nevertheless, the intrinsic physiological mechanism between LBP and impedance remained unclear since the microscopic morphology for different LBP scales was not measured. To date, several studies have investigated the influence of microscopic conditions of biological tissues on bioimpedance, such as the cell morphology (Wang et al., 2015; Song et al., 2018) and electrolyte concentrations in biological tissues (Kumar et al., 2019a,b). Therefore, further work on intrinsic physiological mechanism will be performed in order to provide a solid biophysical fundamental to use EBI for early detection of LBP among helicopter pilots.

## Conclusion

In this study, we explored the feasibility of using EBI technique to detect LBP by means of measuring the lumbar muscle impedance of 72 helicopter pilots and quantitatively analyzing correlation between muscle impedance characteristics and LBP scale. Results showed that for different LBP scales, there existed the significant differences in the modulus of sum of lumbar muscle impedance on both sides, as well as the modulus and phase angle of difference of lumbar muscle impedance on both sides, respectively. These results suggested that EBI has potential to be the daily detection tool in primary medical units to assess the state of the lumbar muscle associated with LBP, so that timely medical intervention could be carried out prior to the observation of apparent symptoms. Future work focusing on optimization of measurement parameters, extraction of impedance features and development of portable impedance measurement system would be designed to improve the practical application of EBI for early detection of LBP among helicopter pilots.

## Data Availability Statement

The original contributions presented in this study are included in the article/supplementary material, further inquiries can be directed to the corresponding authors.

## Ethics Statement

The studies involving human participants were reviewed and approved by the Ethics Committee of Fourth Military Medical University (No. KY20163064-1). The patients/participants provided their written informed consent to participate in this study.

## Author Contributions

HW, JD, CW, ZG, YL, GT, and LY contributed to this research and subsequent manuscript from conception to final preparation. HW, JD, YL, and ZZ helped design, collect, and analyze data. All authors contributed to the article and approved the submitted version.

## Conflict of Interest

The authors declare that the research was conducted in the absence of any commercial or financial relationships that could be construed as a potential conflict of interest.

## Publisher’s Note

All claims expressed in this article are solely those of the authors and do not necessarily represent those of their affiliated organizations, or those of the publisher, the editors and the reviewers. Any product that may be evaluated in this article, or claim that may be made by its manufacturer, is not guaranteed or endorsed by the publisher.
